# The “DeepSeek effect” and the adoption–integration gap of generative artificial intelligence in clinical practice: a national online convenience cross-sectional survey of academic critical care physicians in China

**DOI:** 10.3389/fmed.2026.1875770

**Published:** 2026-06-23

**Authors:** Yuankai Zhou, Wanrong Xiong, Huiying Liu, Qianlin Wang, Hongbo Luo, Yingying Yang, Shengjun Liu, Yun Long

**Affiliations:** 1 Department of Critical Care Medicine, Peking Union Medical College Hospital, Beijing, China; 2 Department of Health Care, Peking Union Medical College Hospital, Beijing, China

**Keywords:** clinical implementation, clinical informatics, critical care medicine, generative artificial intelligence, medical education

## Abstract

**Background:**

The public release of DeepSeek marked a key turning point for generative artificial intelligence (GAI) uptake in China. This study assessed the so-called “DeepSeek effect” on Chinese academic critical care physicians (defined as clinicians with formal concurrent clinical, educational and research duties), comparing changes in self-reported GAI proficiency, clinical practice integration and structured training before and after the model’s launch.

**Methods:**

We conducted a national online convenience cross-sectional study of two independent physician cohorts from Chinese tertiary hospitals: a pre-DeepSeek cohort surveyed in December 2024 (*n* = 456), and a post-DeepSeek cohort surveyed in December 2025 (*n* = 372). Validated questionnaires were used to evaluate GAI knowledge, usage and ethical perceptions. Multivariate logistic regression and exact tests were applied to analyze links between training programs and self-reported professional competence.

**Results:**

Self-reported GAI usage rose significantly from 64.7 to 94.1% after DeepSeek’s release (*p* < 0.001), driven by technical advances of domestic models and improved accessibility. In contrast, formal GAI training participation remained persistently low (13.2% vs. 13.7%, *p* = 0.84). Less than 30% of trained physicians completed structured training, which was strongly associated with enhanced self-reported professional competence (adjusted odds ratio [AOR] = 22.2, 95% confidence interval [CI]: 1.6–305.6, *p* = 0.021). Surveyed physicians also prioritized critical integration skills over basic technical proficiency (OR = 16.3, 95% CI: 3.6–73.3, *p* < 0.001).

**Conclusion:**

The “DeepSeek effect” drove near-universal GAI adoption, but revealed a stark adoption–integration gap with no corresponding gains in self-reported professional competence. Preliminary exploratory data suggest that multidimensional structured training may be a promising associated factor to bridge this gap. GAI implementation efforts must shift from promoting basic uptake to building structured training frameworks for safe, effective, critically appraised clinical integration.

## 1 Introduction

Generative artificial intelligence (GAI) is increasingly used in medicine and medical education (1–3). Critical care medicine is characterized by complex pathologies, urgent decisions, and low error tolerance, where precise individualized diagnosis and treatment are particularly demanding (4, 5). GAI application can enhance accurate diagnoses and individualized therapy in critical care where clinical complexity and urgency require precision (5, 6).

In China, early adoption of international GAI models such as ChatGPT faced substantial structural barriers. These included restricted access, usage costs, and most notably, a mismatch between the models’ linguistic and contextual training and the needs of China’s domestic healthcare system (7, 8). A national survey we conducted in December 2024, during the early phase of GAI uptake among Chinese academic critical care physicians, found that only 64.7% of respondents had used GAI in clinical practice, while just 33.1% had applied the technology to medical education. Most respondents also raised significant ethical concerns regarding over-reliance on GAI outputs and patient data privacy risks (9).

These findings are consistent with global patterns where a persistent adoption-integration gap remains in clinical AI implementation. A 2026 nationwide survey of French cardiologists, known as the INSIGHT-AI study, found that while 63% of professionals used AI in practice, only 7.8% had received formal training (10). Drawing on these international signals, we define the adoption-integration gap as the statistically significant mismatch between the two dimensions outlined above: widespread GAI tool uptake (adoption) and the absence of corresponding improvements in self-reported professional competence (integration). We acknowledge that full institutional-level integration typically involves formal governance, standardized workflow embedding, and systematic quality assurance, which are not measured in the present study. For the purpose of this analysis, we operationally define integration as early individual-level behaviors, including the ability to critically appraise rather than passively accept GAI outputs and self-reported improvements in professional competence. All subsequent references to integration throughout this manuscript follow this explicit operational definition.

In January 2025, the public release of advanced DeepSeek models enabled a major shift in China, providing high-performance, open access, domestically developed, large language models with excellent Chinese ability (11, 12). We call the rapid uptake and incorporation of these local models the “DeepSeek effect” (13), which translates into fewer barriers to adoption and the ability to provide a rapid infusion of high-performance reasoning into clinical workflows (14, 15).

Our study builds on a previous study (9) that quantified the effect of DeepSeek on GAI adoption, trust, and perceived utility among Chinese academic critical care physicians.

This study aims to assess the changes in physician perceptions, quantify the adoption–integration gap emerging from the “DeepSeek effect” and investigate the role of structured GAI training as a potential associated factor in bridging this gap. This population was selected as the study focus because they are the primary early adopters of GAI across clinical, educational, and research workflows, and their practices directly shape institutional GAI governance and training for the broader critical care workforce.

Our findings are intended to support healthcare leaders by offering evidence that can aid in the development of targeted structured GAI training that might improve its clinical implementation and impact.

## 2 Materials and methods

### 2.1 Study design and participants

We conducted an online convenience cross-sectional study analyzing and comparing two separate datasets. In both datasets, the participants were attending, senior academic critical care physicians involved in clinical teaching at major tertiary hospitals across China. Eligible participants were explicitly defined as critical care physicians with ongoing formal responsibilities for clinical patient care, resident medical education, and scientific research in their institutions. In our study, we recruited the physicians via the WeChat professional social media platform.

The two data sets comprised: a pre-DeepSeek cohort surveyed December 2024 (9) prior to the first release on January 15, 2025 of the DeepSeek app; and a post-DeepSeek cohort surveyed from December 17 to December 31, 2025, approximately 1 year later. The anonymous online questionnaire was distributed both during national critical care academic conferences and via nationwide academic WeChat professional groups for critical care physicians across China.

This recruitment strategy covered 31 Chinese provinces. Detailed provincial distribution of respondents is provided in Appendix 1, showing balanced representation across all three major regions: 40.1% eastern, 26.3% central, and 33.6% western China. A STROBE-compliant participant flow diagram is provided as Supplementary Figure S1.

### 2.2 Sample size

We calculated the sample size to ensure adequate statistical precision for our primary outcomes, not to demonstrate probabilistic representativeness of the entire target population. In our post-DeepSeek survey, we used the standard formula for finite populations to calculate our appropriate sample size (16), as was done in the pre-DeepSeek cohort study (9). We based the post-DeepSeek cohort calculation on a pilot survey of 60 participants, which indicated GAI usage rates of approximately 91.6% in medical work, 66.7% in medical education, and 70% in research activities. We adopted the lowest rate (66.7%) for the estimation model. Assuming a 95% confidence level (*Z* = 1.96) and a 5% margin of error, given that no reliable, validated published data are available for the number of eligible academic critical care physicians, we anchored the total eligible target population (*N*) to the published national estimate of 140,000 total critical care physicians in China. This deliberate conservative choice produces a larger minimum sample size requirement than the true eligible population would need, ensuring adequate statistical power. The minimum required number of valid responses was estimated to be 340. We ultimately collected 372 valid responses, exceeding this threshold. All values were rounded to the nearest whole number for practical application.

### 2.3 Data collection instruments

The pre-DeepSeek structured questionnaire had been based on prior validated research (9). Our post-DeepSeek questionnaire retained all the same core items for comparability and included additional questions specific to the use of DeepSeek and other primary domestic models, with minor refinements to some items.

Using the pre-DeepSeek questionnaire as a model, we covered the same five core domains in the post-DeepSeek questionnaire: (1) Demographics; (2) GAI Knowledge & Training; (3) GAI Use in Clinical Practice; (4) GAI Use in Medical Education; and (5) Ethical Perceptions. For the post-DeepSeek questionnaire, we placed specific emphasis on GAI applications in clinical practice, and added supplementary items to assess its use in scientific research workflows (17, 18). Psychometric validation of the tool focused on 18 core Likert-scale items, divided into three pre-specified subscales: Attitudes Toward GAI in Medical Education (8 items), Clinical Support Efficacy of GAI (6 items), and Research Support Functions of GAI (4 items). Categorical demographic and yes/no items were excluded from psychometric testing.

To reduce the risk of response bias, the questionnaire was fully anonymous, with items presented in a random order for each participant. All respondents were explicitly informed that there were no “correct” or “incorrect” answers to the survey questions. Electronic informed consent was obtained via an introductory page that detailed the study’s aims, potential risks and benefits, and the fully voluntary nature of participation; respondents were required to check a mandatory box to confirm consent before proceeding. No personally identifiable information was collected at any point to preserve participant anonymity. Duplicate responses were identified and excluded via truncated IP address tracking, with IP data stored separately from the main study dataset to maintain data integrity.

Content validity was assessed by a panel of six interdisciplinary experts using a four-point relevance scale (three medical education specialists, two clinical chief physicians, and one AI ethics scholar). Appendix 2 presents the final version of the questionnaire.

### 2.4 Definition of structured GAI training

The structured training of GAI was defined post-hoc based-on training content reports. We defined a structured GAI program as one that incorporated three dimensions: (1) Structural: Systematic longitudinal curriculum or hands-on clinical simulation; (2) Safety & Technical: Methods for verifying medical accuracy or identifying GAI-specific risks; and (3) Integration & Ethical: Guidance on clinical workflow integration or coverage of ethical, legal, and policy aspects. GAI training programs lacking any dimension were classified as unstructured.

### 2.5 Data management and statistical analysis

We analyzed the descriptive results in terms of their percentages (counts) for categorical variables and medians (IQR) for ordinal variables. For the between-group comparisons, we used *χ*^2^ tests (or Fisher’s exact test when expected cells were < 5) for categorical items and Mann–Whitney *U* tests for Likert items. We analyzed the multiple-choice items as dichotomous indicators. To address potential confounding from simultaneous policy shifts and the emergence of multiple GAI tools, we used a weighted ranking system (Q15) to isolate the primary drivers of physician behavior. For the drivers of adoption, the weighted ranking was converted into aggregate weighted scores and a relative importance index (RII).

To identify the associations between training and integration outcomes, we constructed multivariate logistic regression models, adjusting for age group; sex; and professional title. Our training analysis comprised two distinct components: first, an effect size analysis comparing participants who received structured training to all other post-DeepSeek participants; second, a descriptive content analysis restricted to the subset of individuals who reported receiving any form of GAI training.

We assessed internal consistency using Cronbach’s alpha and composite reliability (CR). Construct validity was evaluated via confirmatory factor analysis (CFA), convergent validity (average variance extracted [AVE]), and discriminant validity (Fornell–Larcker criterion and the Heterotrait-Monotrait Ratio). A competing model analysis was used to compare a hypothesized three-factor structure with a unidimensional model. For the small structured training subgroup analysis, Fisher’s exact test was used as the gold standard exact statistical method for 2 × 2 contingency tables with small cell counts and potential quasi-separation.

Statistical analyses were performed using IBM SPSS Statistics (version 23.0; IBM Corp., Armonk, NY, USA) and AMOS (version 29; IBM Corp.). Statistical significance was set at a two-tailed *p* value < 0.05. The questionnaire required that all items be answered before submission; thus, no data were missing in the final dataset.

### 2.6 Ethical considerations

The study protocol was approved by the Institutional Review Board of Peking Union Medical College Hospital (I-25ZM0090) and conducted in accordance with the Declaration of Helsinki.

## 3 Results

### 3.1 Instrument validation

The 40-item post-DeepSeek Survey demonstrated sound psychometric properties (see Appendix 3). Internal consistency (Cronbach’s *α* = 0.925–0.959; CR = 0.927–0.963) and convergent validity (AVE > 0.70) both met standard thresholds. Discriminant validity was supported, with the Heterotrait-Monotrait Ratio < 0.86 and inter-factor correlations ranging from 0.672 to 0.805.

The questionnaire demonstrated excellent content validity, as evidenced by a scale-level content validity index (S-CVI/Ave) of 0.986. Confirmatory factor analysis further confirmed acceptable model fit (*χ*^2^/df = 7.948, comparative fit index [CFI] = 0.911, standardized root mean square residual [SRMR] = 0.037), supporting the reliability of the instrument.

### 3.2 Baseline characteristics, knowledge and use of GAI

The pre-DeepSeek study included 456 participants, and our post-DeepSeek study 372 participants. Thus, in total, 828 Chinese academic critical care physicians were surveyed.

There was good balance across the demographic and professional characteristics in both survey groups (Table 1), with the comparisons showing negligible effect sizes (Cohen’s *d* < 0.1) (Supplementary Table S1).

**Table 1:** Baseline characteristics and GAI literacy of critical care physicians (Pre-DeepSeek vs. Post-DeepSeek).

Variables	Total (*n* = 828)	Pre-DeepSeek (*n* = 456)	Post-DeepSeek (*n* = 372)	*p* value
Age, y	36 (31–40)	36 (31–40)	37 (32–41)	0.190
Male, *n* (%)	466 (56.3)	263 (57.7)	203 (54.6)	0.370
Tertiary hospital, *n* (%)	828 (100)	456 (100)	372 (100)	>0.999
Professional title, *n* (%)				
Attending physician	524 (63.3)	300 (65.8)	224 (60.2)	0.111
Associate chief physician	230 (27.8)	123 (27.0)	107 (28.8)	0.066
Chief physician	74 (8.9)	33 (7.2)	41 (11.0)	0.681
Using GAI, *n* (%)	645 (77.9)	295 (64.7)	350 (94.1)	<0.001
Using GAI before January 2025, *n* (%)	–	–	95 (25.5)	–
Participated GAI Training, *n* (%)	111 (13.4)	60 (13.2)	51 (13.7)	0.838
Understanding of GAI^†^	2 (2–3)	2 (2–3)	2 (2–3)	<0.001
Change in GAI use frequency since January 2025, *n* (%)				
Increased	–	–	350 (94.1)	
Decreased	–	–	22 (5.9)	
Remained unchanged	–	–	0 (0)	
Using GAI in medical education, *n* (%)	347 (41.9)	151 (33.1)	196 (52.7)	<0.001
Which GAI model do you use most frequently				
DeepSeek and related GAI	–	0 (0)	120 (34.3)	<0.001
Other Chinese GAI models	–	264 (89.5)	222 (63.4)	<0.001
Other U.S. GAI models	–	31 (10.5)	8 (2.2)	<0.001
Frequency if using GAI (*n* = number of using GAI)				
Daily, *n* (%)	182 (28.2)	63 (21.4)	119 (34.0)	< 0.001
Weekly, *n* (%)	273 (42.3)	106 (35.9)	167 (47.7)	0.003
Monthly, *n* (%)	126 (29.5)	126 (42.7)	64 (18.3)	< 0.001

Values are given as median (interquartile range) or number (percentage).

^†^Understanding of GAI: Very knowledgeable = 4; Moderately knowledgeable = 3, Slightly knowledgeable = 2, Not knowledgeable at all = 1.

*p* value: Comparisons between the pre-DeepSeek group and the post-DeepSeek group were performed using the Mann–Whitney *U* Test or Chi-square test.

GAI, Generative Artificial Intelligence.

**Figure 1:**
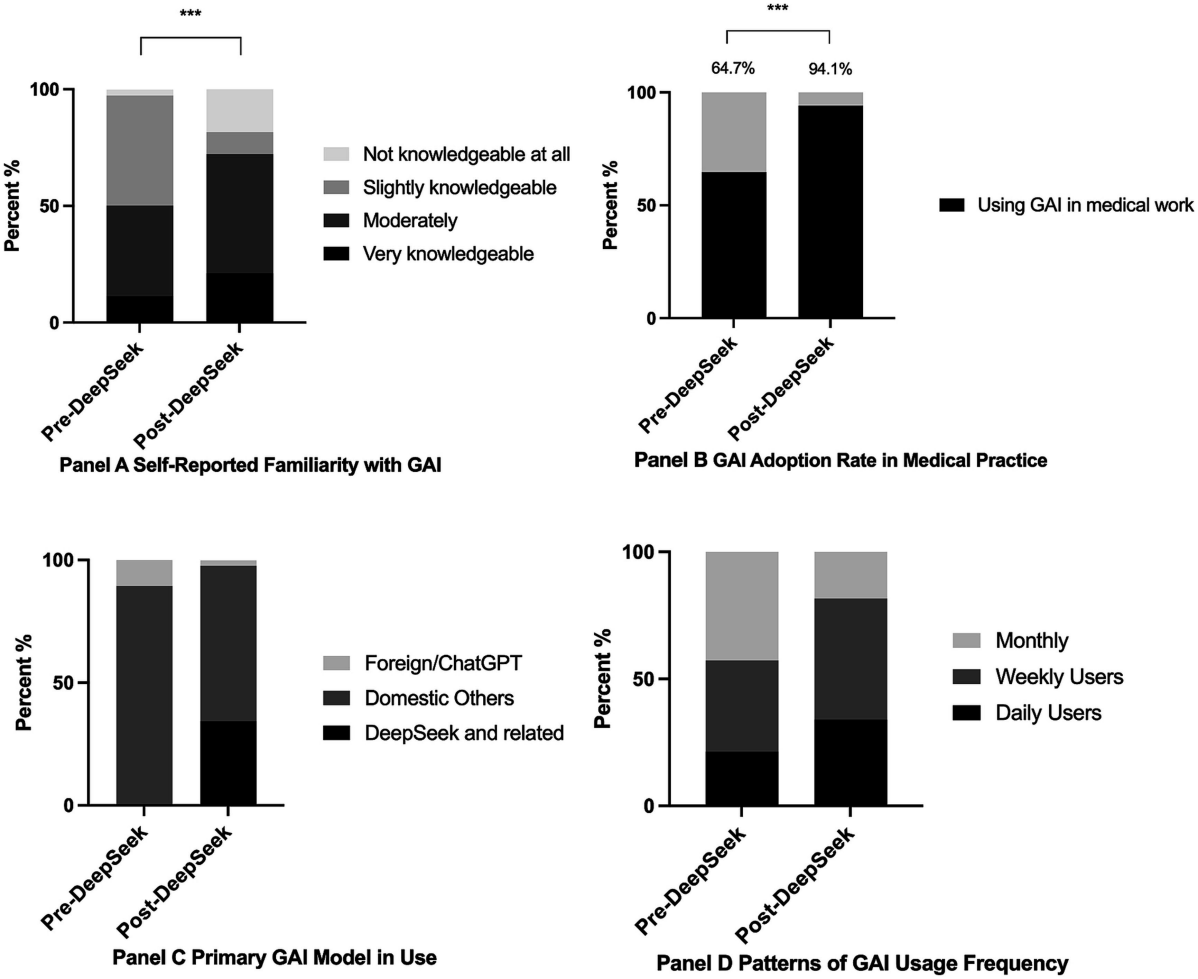
Trends in GAI Adoption and Usage Patterns Before and After DeepSeek Release. Composite figure displaying changes in GAI-related behaviors among Chinese critical care physicians before (Pre-DeepSeek) and after (Post-DeepSeek) the public release of the DeepSeek model. **(Panel A)** Self-reported familiarity with GAI, categorized as “Not knowledgeable at all”, “Slightly knowledgeable”, “Moderately knowledgeable”, or “Very knowledgeable.” There was a significant increase in self-reported knowledge (*p <* 0.001, Wilcoxon signed-rank test). **(Panel B)** Rate of using GAI in medical work. Adoption increased from 64.7 to 94.1% (*p <* 0.001). **(Panel C)** Primary GAI model used. Use of DeepSeek and related domestic models rose significantly, while usage of foreign models (e.g., ChatGPT) declined. **(Panel D)** Frequency of GAI use. Daily and weekly users increased substantially post-DeepSeek, indicating deeper integration into clinical workflows. All panels show stacked bar charts of percentages (%), with sample sizes balanced across cohorts (*n =* 456 pre-DeepSeek; *n =* 372 post-DeepSeek). The denotes statistical significance at *p <* 0.001.

However, we saw a marked upsurge in GAI engagement among our post-DeepSeek cohort participants. Comparing the physician responses in the pre- and post- surveys, we found that GAI user adoption increased from 64.7 to 94.1% (*p <* 0.001), and its usage in medical education rose from 33.1 to 52.7% (*p <* 0.001). We also noted significant changes in the type of GAI being used. In the post-DeepSeek survey, the combined usage rate of DeepSeek and related domestic GAI models reached 34.3%, with usage of foreign GAI models decreasing from 10.5% in the pre-DeepSeek cohort to 2.2% in the post survey (Table 1 and Figure 1C).

### 3.3 Drivers of the surge in GAI usage

We identified the drivers of increased GAI adoption among the post-DeepSeek cohort using a weighted ranking analysis (Table 2). The most influential factor was technological advancements in domestic models (e.g., enhanced reasoning capabilities and improved Chinese language support), which represented 37.2% on the RII and was identified as the primary driver by 50.0% of the GAI users (*n =* 175). The second most important factor was improved model accessibility and usability (e.g., no network restrictions, faster response speed, and reduced costs), representing 31.6% of the RII. Together, these two factors accounted for 68.8% of the cumulative RII, highlighting their dominant role in driving nationwide GAI adoption.

**Table 2:** Drivers of the Post-DeepSeek usage surge: a weighted ranking analysis (Q15).

Rank	Factors driving increased GAI usage	Weighted score	Primary driver (Rank 1) (n)	Primary driver (Rank 1) (%)	Relative importance index (RII)
1	Technological advancement of domestic models (e.g., enhanced reasoning, improved Chinese language support)	781	175	50.0%	37.2%
2	Improved accessibility and ease of use (e.g., no network restrictions, high response speed, lower costs)	664	110	31.4%	31.6%
3	Objective growth in clinical and research workloads	382	40	11.4%	18.2%
4	Data security and regulatory compliance (e.g., localized deployment, privacy protection)	135	15	4.3%	6.4%
5	Influence from professional peers and academic media (e.g., recommendations, expert opinions)	92	8	2.3%	4.4%
6	Institutional policy guidance or infrastructure improvements	46	2	0.6%	2.2%
Total		2,100	350	100.0%	100%

This table quantifies the primary motivators for GAI adoption through a weighted ranking system. Respondents prioritized their top three driving factors, with weights assigned as 3, 2, and 1 for Ranks 1, 2, and 3, respectively. The Weighted Score (S_i_) for each factor was calculated using the formula: S_i_ = (n_i,1_ × 3) + (n_i,2_ × 2) + (n_i,3_ × 1), where n_i,j_ represents the number of respondents who ranked factor i at position j. The Relative Importance Index (RII): represents the proportion of each factor’s weighted score relative to the aggregate score of all factors (S_total_ = 2,100). A higher RII signifies a more substantial contribution to the behavioral shift toward increased GAI integration observed in the Post-DeepSeek cohort.

GAI, Generative Artificial Intelligence.

### 3.4 Perceived efficacy of GAI

**Figure 2:**
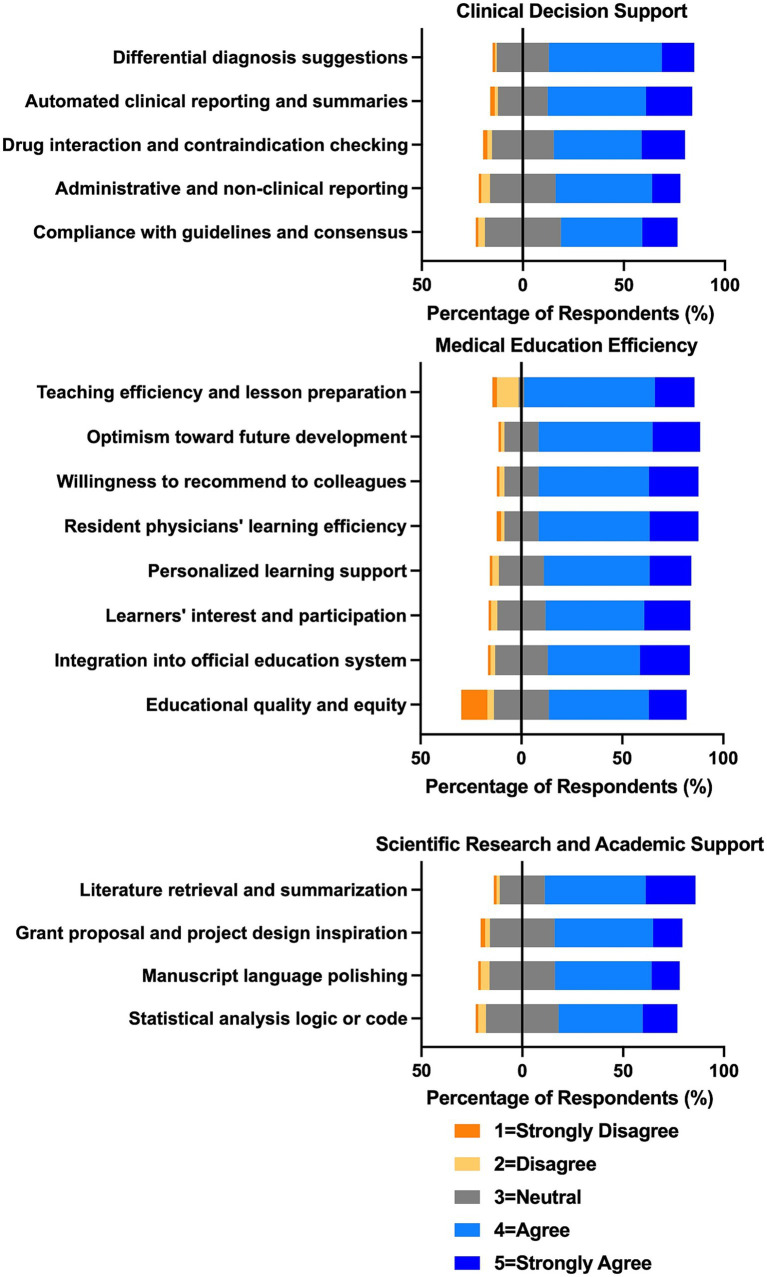
Perceived efficacy of Generative AI across clinical, educational, and research domains. horizontal stacked bar chart illustrating the perceived utility of generative AI (GAI) in three key domains among Post-DeepSeek cohort physicians (*n =* 372). Responses were collected on a 5-point Likert scale: 1 = Strongly Disagree, 2 = Disagree, 3 = Neutral, 4 = Agree, 5 = Strongly Agree. Colors represent agreement levels: orange (strong disagreement), yellow (disagreement), gray (neutral), blue (agreement), dark blue (strong agreement). Clinical Decision Support: High agreement (> 80%) on usefulness for differential diagnosis suggestions, automated reporting, drug interaction checking, and guideline compliance. Medical Education Efficiency: Strong positive perception regarding teaching efficiency, resident learning, and willingness to recommend GAI to peers. However, concerns about educational quality and equity remain evident (moderate disagreement). Scientific Research and Academic Support: Over 85% agreed or strongly agreed that GAI is useful for literature retrieval, abstract generation, grant proposal inspiration, and manuscript polishing. This figure demonstrates broad acceptance of GAI in clinical and research contexts, with nuanced attitudes in education—highlighting both opportunities and challenges for its integration.

In the post-DeepSeek cohort, respondents provided detailed evaluations of GAI utility for clinical, educational, and research practice (Figure 2, Supplementary Table S2). In clinical decision support, GAI’s differential diagnosis suggestions and automated clinical documentation received the highest marks, with over 80% of the respondents agreeing or strongly agreeing with these practical applications. In medical education, although improved teaching efficiency was recognized, a considerable proportion of respondents remained concerned about the potential impacts of GAI on educational quality and equity. In scientific research, the most valued GAI functions were literature retrieval and summarization (> 85% positive rate), followed by support for grant proposal inspiration and manuscript polishing.

### 3.5 Trust in GAI and ethical perceptions

Across the two cohorts, there were no changes in the physicians’ recognition of issues such as knowledge cutoffs, hallucinations, inter-model inconsistency, data-driven performance variability, and reliance on unverified training data (all *p >* 0.05). Similarly, major ethical concerns showed no statistically significant changes (all *p >* 0.05) (Table 3).

**Table 3:** Perceptions of GAI constraints, ethical concerns, and trust: a Pre- and Post-DeepSeek comparison.

Domain and evaluated items	Pre-DeepSeek (*n*, %)	Post-DeepSeek (*n*, %)	*χ*^2^/*Z*	*p* value^#^
I. Perception of GAI technical constraints (Q17, Multi-choice)^a^	(*n* = 251)	(*n* = 350)		
Data-driven performance variability across models	154 (61.4)	202 (57.7)	0.802	0.370
GAI knowledge cutoff and temporal limitations	148 (59.0)	206 (58.9)	0.001	0.979
Inter-model inconsistencies in logic and style	133 (53.0)	201 (57.4)	1.167	0.280
Speculative outputs and “hallucination” phenomena	126 (50.2)	183 (52.3)	0.255	0.614
Use of unverified public training data	141 (56.2)	216 (61.7)	1.860	0.173
II. Top ethical and safety concerns (Q34, Multi-choice)^a^	(*n* = 456)	(*n* = 372)		
Over-reliance on automated assistance	394 (86.4)	321 (86.3)	0.002	0.962
Data privacy and confidentiality risks	380 (83.3)	294 (79.0)	2.503	0.114
Academic misconduct and integrity issues	353 (77.4)	268 (72.0)	3.150	0.076
Erosion of cognitive and clinical judgment	350 (76.8)	305 (82.0)	3.397	0.065
III. Trust calibration under uncertainty (binary)	(*n* = 456)	(*n* = 372)		
Trust GAI when it contradicts clinical judgment (“Yes”)	230 (50.4)	157 (42.2)	5.580	0.018*
Trust GAI when no prior clinical judgment exists (“Yes”)	259 (56.8)	243 (65.3)	6.237	0.013*
Willingness to recommend GAI to colleagues (“Yes”)	413 (90.6)	356 (95.7)	9.907	0.002*
Perceived GAI impact on self-reported professional competence (ordinal data)			−1.593	0.161
Increase = 3	278 (61.0)	209 (56.2)		
No impact = 2	161 (35.3)	140 (37.6)		
Decrease = 1	17 (3.7)	23 (6.2)		

^a^For multi-choice questions (Q17, Q34), percentages represent the proportion of respondents who selected the item; the total may exceed 100%.

^*^p < 0.05.

^#^Statistical comparisons between the two groups were performed using the chi-square test for categorical variables and the Mann–Whitney *U* test for ordinal data.

However, trust in GAI outputs revealed significant scenario dependence. When outputs conflicted with personal clinical judgments, trust decreased from 50.4% (pre-DeepSeek) to 42.2% (post-DeepSeek) (*p* = 0.018); conversely, in the absence of clinical judgments, trust increased from 56.8% (pre-DeepSeek) to 65.3% (post-DeepSeek) (*p =* 0.013).

Notably, in the post-survey, physicians were more likely to recommend GAI to peers (90.6% vs. 95.7%, *p =* 0.002).

### 3.6 Predictors of GAI integration in education and self-reported professional competence

In the post-DeepSeek survey, multivariable logistic regression identified familiarity with GAI (adjusted odds ratio [AOR] = 1.29; 95% CI: 1.08–1.55, *p =* 0.006) and usage frequency (AOR = 1.29; 95% CI: 1.06–1.57, *p =* 0.012) as predictors of higher integration into medical education (Table 4, Model 1).

**Table 4:** Logistic regression analysis of predictors for GAI integration in medical education and self-reported professional competence (*N* = 828).

Predictors (variables)	Univariate (crude OR)	*p* value	Multivariate (adjusted OR)	*p* value
Model 1: Integration into medical education^a^				
Group (Ref: Pre–Post)	2.25 (1.70–2.98)	0.209		
Familiarity with GAI (1–4 scale) ^†^	1.26 (1.07–1.49)	0.005*	1.29 (1.08–1.55)	0.006*
GAI usage frequency (1–3 scale) ^#^	1.27 (1.05–1.55)	0.016*	1.29 (1.06–1.57)	0.012*
Participated GAI training	1.27 (0.95–1.70)	0.105		
Age	1.01 (0.99–1.03)	0.244		
Gender	0.84 (0.63–1.11)	0.216		
Academic title	1.02 (0.83–1.25)	0.856		
Model 2: Perceived effect on self-reported professional competence^b^			-	
Group (Ref: Pre–Post)	0.80 (0.61–1.05)	0.111		
Familiarity with GAI (1–4 scale)	0.99 (0.85–1.16)	0.932		
GAI usage frequency (1–3 scale)	1.20 (0.99–1.46)	0.063	1.21 (1.00–1.48)	0.055
Participated GAI training	1.52 (1.14–2.04)	0.005*	1.98 (1.37–2.86)	< 0.001
Age	1.00 (0.98–1.02)	0.747		
Gender	1.03 (0.78–1.36)	0.818		
Academic title	1.05 (0.85–1.28)	0.662		

^a^Model 1 used binary logistic regression. Dependent variable: “Have you used GAI in medical education?” (0 = No, 1 = Yes) (Q).

^b^Model 2 applied ordered logistic regression to accommodate the 3-point scale of perceived effect on self-reported professional competence (1 = decrease, 2 = no impact, 3 = increase) (Q18).

^†^Familiarity with GAI: Not knowledgeable at all = 1; Slightly knowledgeable = 2, Moderately knowledgeable = 3, Very knowledgeable = 4.

#: GAI Usage Frequency: Monthly = 1; Weekly = 2, Daily = 3.

GAI, Generative Artificial Intelligence.

There was no statistically significant difference in the perceived effect on self-reported professional competence between the two cohorts (*Z* = −1.593, *p =* 0.16) (Table 3). This lack of improvement in competence despite the surge in adoption clearly delineates the adoption–integration gap.

General participation in any GAI training was associated with nearly double the odds of reporting enhanced self-reported professional competence (AOR = 1.98, 95% CI: 1.37–2.86, *p <* 0.001) (Table 4).

### 3.7 Structured training: prevalence and association with self-reported professional competence

Despite near-universal GAI adoption (94.1%, 350/372) among post-DeepSeek respondents, participation in formal training remained disproportionately low at only 13.7% (*n* = 51). Of these, a mere 29.4% (*n* = 15) participated in programs meeting our predefined criteria for structured training, while the remaining 70.6% (*n* = 36) received unstructured, self-directed training (Table 5).

**Table 5:** Composition of GAI training content and multivariable-adjusted association between structured training and self-reported professional competence (participated GAI training in Post-DeepSeek Cohort, *n* = 51).

Variable/analysis item	Total sample, *n* (%)	Structured training group (*n* = 15)^a^	Non-structured training group (*n* = 36)	Statistic (*χ*^2^)	*p* value	aOR^e^ (95% CI)
GAI training content reported^b^						
(A) Systematic/longitudinal curriculum, *n* (%)	18 (35.3)	10 (66.7)	8 (22.2)	9.158	0.002	–
(B) Hands-on clinical practice/simulation, *n* (%)	29 (56.9)	15 (100.0)	14 (38.9)	16.12	<0.001	–
(C) Methods for verifying medical accuracy of outputs, *n* (%)	3 (5.9)	3 (20.0)	0 (0.0)	7.650	0.006	–
(D) Identification of GAI-specific risks, *n* (%)	19 (37.3)	15 (100.0)	4 (11.1)	35.79	<0.001	–
(E) Guidance on clinical-workflow integration, *n* (%)	3 (5.9)	0 (0.0)	3 (8.3)	1.328	0.249	–
(F) Ethical/legal/institutional-policy coverage, *n* (%)	18 (35.3)	15 (100.0)	3 (8.3)	38.96	<0.001	–
(G) Focused only on GAI basic tool functions, *n* (%)	21 (41.2)	NA	21 (58.3)	–	–	–
(H) Do not recall the specific content, *n* (%)	4 (7.8)	NA	4 (11.1)	–	–	–
Met “structured training” definition, *n* (%)^a^	15 (29.4)	15 (100.0)	0 (0.0)	–	–	–
Association of structured training with Self-reported professional competence^c^					0.020^d^	22.2 (1.6–305.6)^f^

^a^“Structured training” was defined as training that included content from all three dimensions: (1) Structural (item A or B); (2) Safety and Technical (item C or D); and (3) Integration and Ethical (item E or F). This descriptive content analysis is restricted exclusively to the 51 post-DeepSeek participants who reported receiving any GAI training (structured *n* = 15, unstructured *n* = 36). Note: This is a separate analysis from the regression model presented in Figure 3, which compares structured training (*n* = 15) against all other post-DeepSeek participants (*n* = 357) to assess effect size.

^b^This section includes only respondents who answered “Yes” to “Have you participated in any GAI training?” in Post-DeepSeek Cohort (*n* = 51). Percentages are calculated based on this subsample.

^c^“Enhancement of self-reported professional competence” was derived from the dependent variable in the ordinal regression model (Table 4, Model 2). For display, responses of “increase” were categorized as “Yes,” and “no impact/decrease” as “No”.

^d^Association assessed via ordinal logistic regression, adjusted for gender, age, and professional title. Results are presented as adjusted odds ratios (aOR) with 95% confidence intervals.

^e^Adjusted odds ratio.

^f^Due to the small sample size in the structured training group (*n* = 15), the aOR should be interpreted as exploratory.

Multivariate analysis adjusted for demographic factors revealed a significant association between structured training and perceived improvement in self-reported professional competence (AOR = 22.2, 95% CI: 1.6–305.6, *p =* 0.021). The raw contingency table and Fisher’s exact test results are presented in Supplementary Table S4. However, it is crucial to emphasize that the extremely wide CI resulting from the small sample size (*n =* 15) points to substantial statistical uncertainty, and this finding should be interpreted as hypothesis-generating rather than definitive proof of causal efficacy.

### 3.8 Impact of structured training on core GAI use competencies

**Figure 3:**
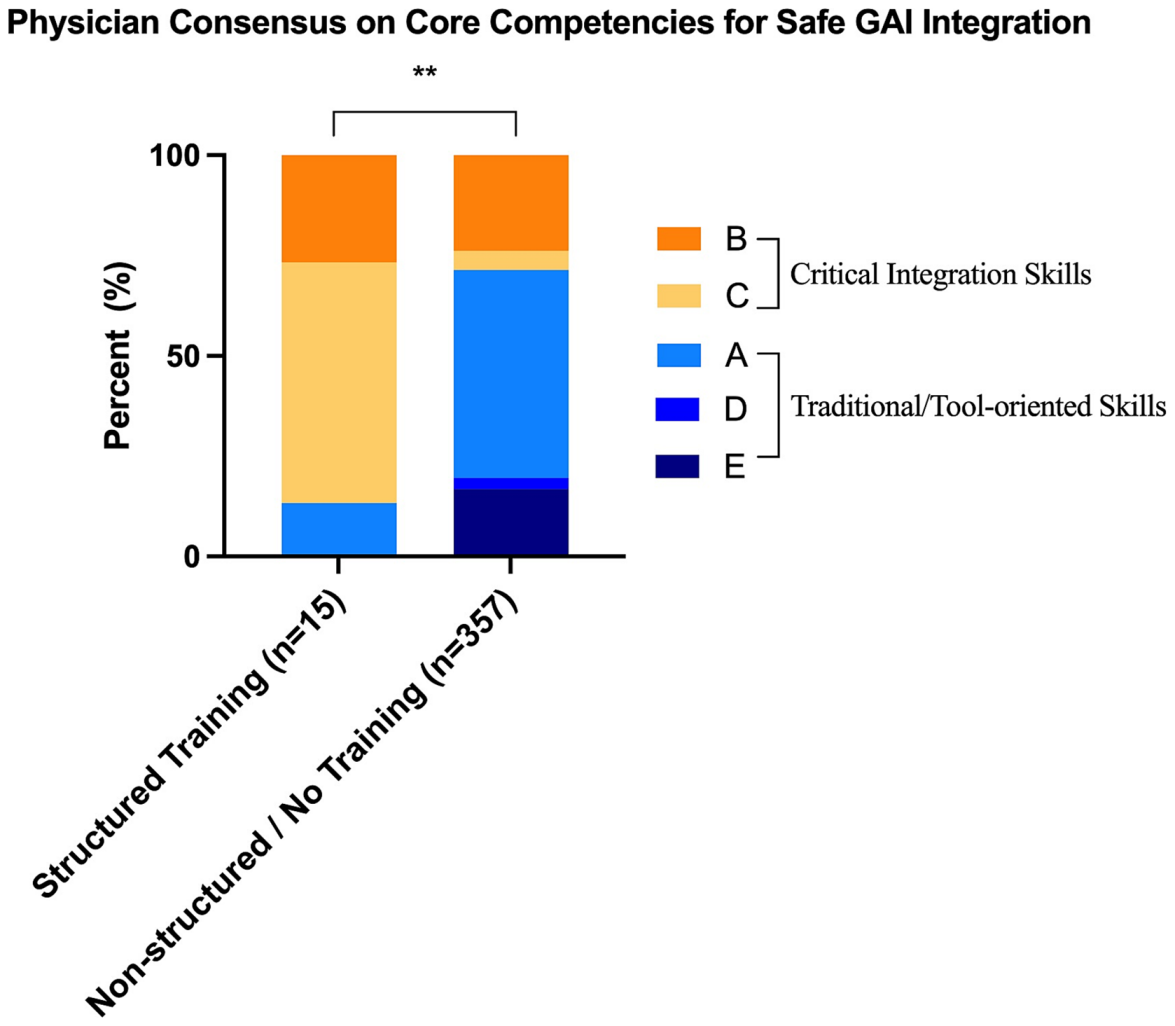
Physician Consensus on Core Competencies for Safe GAI Integration. Stacked bar chart showing the distribution of physician-reported core competencies deemed most important for safe integration of GAI in critical care, stratified by exposure to structured training. Respondents were asked to select one primary competency from a list including: (A) Technical operation; (B) Critical appraisal of outputs; (C) Clinical reasoning integration; (D) Prompt engineering; (E) Data privacy awareness. The categories are grouped into Traditional/Tool-oriented Skills (A, D, E) and Critical Integration Skills (B, C). Among physicians who received structured training (*n =* 15), 86.7% prioritized critical integration skills (B, C), compared to only 28.6% among those without such training (*n =* 357). The difference was statistically significant (logistic regression, *p <* 0.001), indicated by ^**^ above the bars. Among physicians who received structured training (*n* = 15), 86.7% prioritized critical integration skills (B, C), compared to only 28.6% among those without such training (*n* = 357). The difference was statistically significant (logistic regression, *p* < 0.001), indicated ^**^ by above the bars. This shift highlights that structured training may promote a paradigm shift from passive tool use to active cognitive engagement with GAI outputs.

Structured training appeared to significantly influence physicians’ perceptions of core GAI-related competencies (Supplementary Table S3; Figure 3). When asked about the important physician skills for applying GAI in critical care, 86.7% (13/15) of the physicians with structured training identified critical integration skills (critical appraisal or clinical reasoning integration) as the primary competency, compared to only 28.6% (102/357) of those without such training. This difference was statistically significant (OR = 16.3, 95% CI: 3.6–73.3; *p <* 0.001) (Figure 3). The implication is that structured training shifts physicians’ self-assessment from users of GAI to experts in reviewing AI outputs.

## 4 Discussion

### 4.1 Principal findings

Among Chinese academic critical care physicians, the “DeepSeek effect” coincided with a sharp rise in GAI adoption, from 64.7 to 94.1% (*p <* 0.001). Despite near-universal tool use, formal GAI training remained below (13.7%), and self-reported professional competence showed no meaningful improvement over the study period. This empirical observation allowed us to formally define and quantify the adoption-integration gap, a phenomenon that had previously been only anecdotally described in the literature. Notably, the integration assessed in this study reflects only an early individual-level stage of GAI implementation, rather than the full integration involving defined learning objectives, supervised use, workflow embedding, assessment, governance and quality assurance.

Increased GAI utilization was driven mainly by technical improvements in domestic large language models and greater system accessibility, not institutional training or policy changes. This pattern of rapid, near-universal uptake aligns with innovation diffusion theory (19, 20). Among the small subset of providers who completed structured, multidimensional training, self-assessed competence improved significantly (*p =* 0.021). These individuals also shifted their focus from basic tool operation to critical appraisal and clinical reasoning integration (*p <* 0.001). Trust in GAI outputs varied by clinical context: higher when no prior diagnostic judgment existed, and lower when model outputs contradicted existing clinical decisions.

The pre–post surveys revealed no significant difference in subjective ratings of GAI’s impact on self-reported professional competence (Table 3), suggesting that mere tool accessibility does not equate to GAI proficient utilization and integration within clinical practice.

We find that GAI formal education might be a significant associated factor for bridging this adoption–integration gap. Although GAI familiarity and usage frequency predict integration into medical education, only multidimensional structured training reveals a strong association with enhanced self-reported professional competence. This confirms our hypothesis that to transition from GAI adoption to integration, training must evolve from basic technical instruction to structured faculty development focusing on critical appraisal and clinical reasoning. Although the association (AOR = 22.2) needs to be interpreted with caution as the small subgroup size, the overall trend is clear.

Our findings prompt a primary hypothesis: to facilitate academic critical care physicians’ effective integration of GAI into their medical practice, GAI training content needs to extend beyond technical skill instruction to include content accuracy verification and the application of clinical reasoning. A GAI competency-structured training framework (defined in Table 5) can shift physician focus from just applying GAI models to critically examining, integrating, and benefiting from their outputs.

These results are consistent with the INSIGHT-AI France study (10), which also found formal training to be the single most important driver of both regular AI adoption and clinician confidence in AI outputs. This parallel observation across different countries and clinical specialties confirms that the gap is not unique to critical care medicine in China, but represents a common global challenge. Building on these international findings, our study further proposes a clear three-dimensional operational definition of structured training and quantifies its unique value over basic unstructured formal training.

This study also reveals a context-dependent pattern of trust in GAI among the Chinese academic critical care physicians we surveyed. Trust was significantly higher (65.3%) when GAI was used in scenarios lacking preexisting clinical judgments; whereas it was notably lower (42.2%) when the outputs conflicted with established judgments. The implication is that physicians perceive GAI as a supplementary cognitive resource while maintaining a degree of caution regarding potential inaccuracies. This finding is consistent with the prevailing concern that GAI may diminish critical thinking (77.2%); a concern also expressed by the international medical community (21).

From the above analysis, the following recommendations could be proposed:

1. GAI Training Program Development and Evaluation: Educational curricula should explore and evaluate structured GAI training frameworks, particularly incorporating multidimensional training content that our exploratory findings suggest may enhance self-reported professional competence.
2. Capability-Based Assessment: Evaluation criteria for integrating GAI into medical work should shift from technical proficiency to critical content review and contextualized clinical reasoning support.
3. Institutional Protocol Development: Healthcare institutions should establish clear governance protocols, defining appropriate GAI use cases and distinguishing the roles in “AI-assisted” versus “doctor-led” decision-making.
4. Usage Guidelines Establishment: Healthcare institutions should ensure that GAI usage by healthcare professionals does not harm patients or result in the disclosure of patient data or impact privacy.

### 4.2 Limitations

Our study includes several limitations. First, the analysis of the effect of structured training relies on a very small subgroup (*n =* 15), resulting in wide CI and statistical instability. No formal moderation analysis was performed. Thus, the findings on the association between GAI structured training and self-reported professional competence should be considered strictly hypothesis-generating only. Second, the cross-sectional design precludes definitive causal inference, although the pre–post analyses provide strong evidence for a temporal association. Third, this study used an online convenience sampling strategy via WeChat professional groups and national academic conferences, which likely overrepresents digitally active and academically engaged physicians. To preserve participant anonymity, we did not collect specific hospital names, and an exact response rate could not be calculated. The 94.1% post-DeepSeek GAI adoption rate therefore likely overestimates the true national prevalence. Generalizability is further limited to academic critical care physicians in tertiary hospitals; findings may not apply to junior residents, physicians without teaching/research duties, or those in non-tertiary settings. Nevertheless, our core finding of an adoption-integration gap remains valid, and this mismatch would likely be even larger in less digitally engaged populations. Fourth, the anonymous design prevented tracking any individual-level changes. Future studies should adopt a prospective cohort design to evaluate the long-term impact of GAI integration on clinical and educational outcomes. Finally, all assessments of generative AI-related competence in this study were based entirely on self-reported subjective perceptions, and no objective measures were included.

## 5 Conclusion

The “DeepSeek effect” in 2025 propelled GAI adoption to near-universal levels among Chinese academic critical care physicians. However, this surge did not translate into equivalent depth of GAI-medical integration. A significant adoption–integration gap persists, characterized by high adoption but a lack of perceived improvement in self-reported professional competence. Our exploratory findings suggest that multidimensional structured training may serve as a vital associated factor to bridge this gap, facilitating a shift from passive tool usage to active, critical collaboration with GAI, thereby enhancing integration into routine clinical practice. As GAI becomes embedded in clinical infrastructure, the focus of GAI education and clinical implementation must pivot from promoting adoption toward ensuring safe, critical, and effective GAI-medical integration through multidimensional structured training frameworks.

## Data Availability

The raw data supporting the conclusions of this article will be made available by the authors, without undue reservation.
