# Supplementary material for: The “DeepSeek effect” and the adoption–integration gap of generative artificial intelligence in clinical practice: a national online convenience cross-sectional survey of academic critical care physicians in China
Source: Front Med (Lausanne). 2026 Jun 23;13:1875770. doi: 10.3389/fmed.2026.1875770 (PMC13337403; doi:10.3389/fmed.2026.1875770)
Supplement: Supplementary file 1 [file Supplementary_file_1.DOCX]

**Figure S1 STUDY FLOW DIAGRAM**


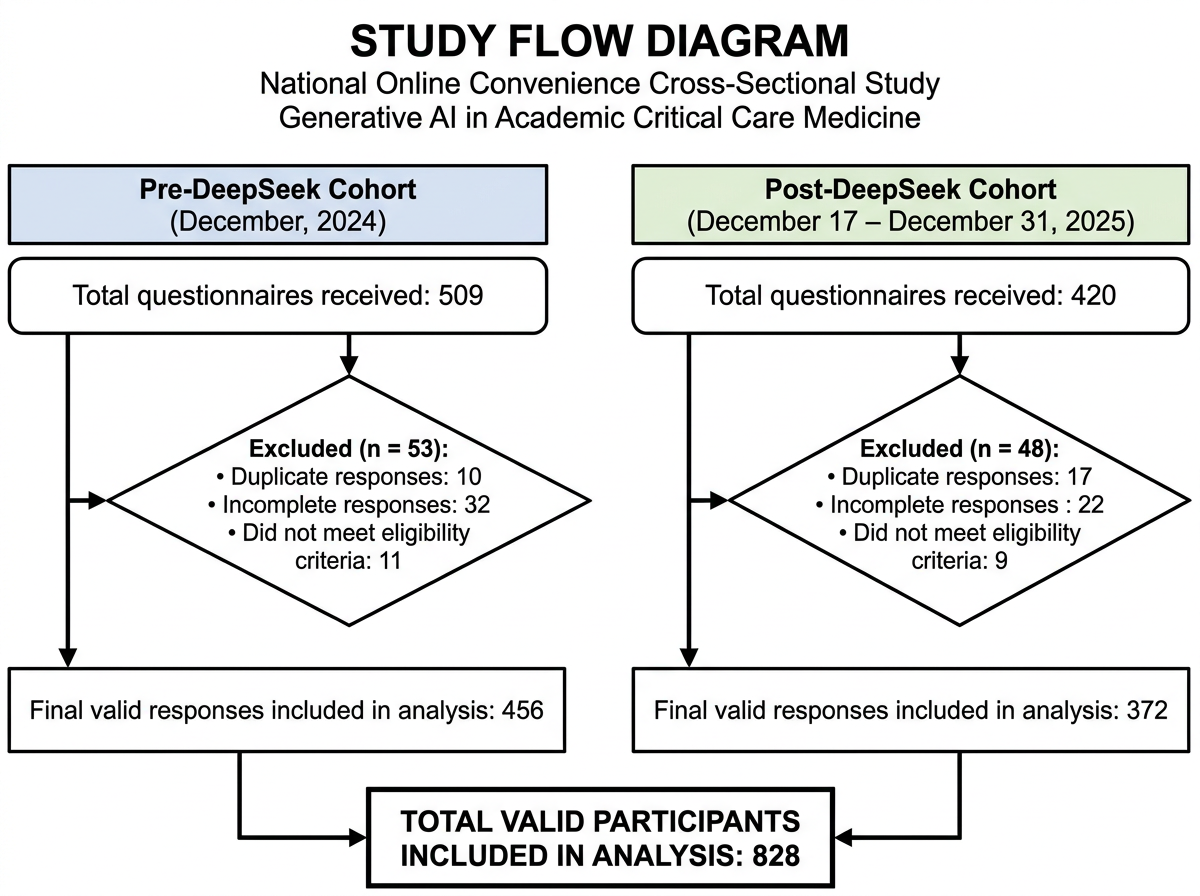


| **Table S1 Pre-DeepSeek vs. Post-DeepSeek Effect size measure** | | |
| --- | --- | --- |
| Variable | S^2^ pooled | Cohen's *d* value |
| Gender | 0.246 | 0.063 |
| Age | 54.232 | 0.038 |
| Professional Title | 0.449 | 0.029 |

**Table S2: Detailed Evaluation of GAI Utility in Medical Education, Clinical Practice, and Research among Chinese Critical Care academic physicians**

(Post-DeepSeek Cohort, N=372)

| Domain & Evaluated Items | n (%) | | | | | Positive Rate^*^, (%) |
| --- | --- | --- | --- | --- | --- | --- |
|  | 1 | 2 | 3 | 4 | 5 |  |
| Clinical Decision Support |  |  |  |  |  |  |
| Differential diagnosis suggestions | 5(1.3) | 3(0.8) | 96(25.8) | 208(55.9) | 60(16.1) | 55.9 |
| Automated clinical reporting and summaries | 8(2.2) | 6(1.6) | 92(24.7) | 181(48.7) | 85(22.8) | 48.7 |
| Drug interaction and contraindication checking | 8(2.2) | 8(2.2) | 114(30.6) | 162(43.5) | 80(21.5) | 43.5 |
| Administrative and non-clinical reporting | 5(1.3) | 12(3.2) | 86(23.1) | 186(50.0) | 83(22.3) | 47.8 |
| Compliance with guidelines and consensus | 5(1.3) | 12(3.2) | 140(37.6) | 150(40.3) | 65(17.5) | 40.3 |
| Medical Education Efficiency |  |  |  |  |  |  |
| Teaching efficiency and lesson preparation | 8(2.2) | 40(10.8) | 10(2.7) | 241(64.8) | 73(19.6) | 84.4 |
| Optimism toward future development | 5(1.3) | 6(1.6) | 63(16.9) | 210(56.5) | 88(23.7) | 80.2 |
| Willingness to recommend to colleagues | 5(1.3) | 9(2.4) | 63(16.9) | 204(54.8) | 91(24.5) | 79.3 |
| Resident physicians' learning efficiency | 8(2.2) | 6(1.6) | 63(16.9) | 205(55.1) | 90(24.2) | 79.3 |
| Personalized learning support | 5(1.3) | 12(3.2) | 83(22.3) | 195(52.4) | 77(20.7) | 73.1 |
| Learners' interest and participation | 5(1.3) | 11(3.0) | 89(23.9) | 182(48.9) | 85(22.8) | 71.8 |
| Integration into official education system | 5(1.3) | 8(2.2) | 97(26.1) | 170(45.7) | 92(24.7) | 70.4 |
| Educational quality and equity | 5(1.3) | 12(3.2) | 101(27.2) | 184(49.5) | 70(18.8) | 68.3 |
| Scientific Research and Academic Support |  |  |  |  |  |  |
| Literature retrieval and summarization | 5(1.3) | 16(4.3) | 121(32.5) | 178(47.8) | 52(14.0) | 74.7 |
| Grant proposal and project design inspiration | 8(2.2) | 9(2.4) | 119(32.0) | 182(48.9) | 54(14.5) | 63.4 |
| Manuscript language polishing | 5(1.3) | 21(5.6) | 113(30.4) | 170(45.7) | 63(16.9) | 61.8 |
| Statistical analysis logic or code | 5(1.3) | 14(3.8) | 134(36.0) | 155(41.7) | 64(17.2) | 58.9 |

*Positive Rate: Percentage of respondents scoring 4 (Agree) or 5 (Strongly Agree)

**Table S3: Physician Consensus on Core Competencies for GAI Integration and Insights from GAI Trained Subgroups in Post-DeepSeek Cohort**

| Analysis and Competency Category | Structured Training Group (n=15) | Non-structured/No Training Group (n=357) | OR (95% CI) | *p* value ^a^ |
| --- | --- | --- | --- | --- |
| **Primary Analysis: Binary Comparison** |  |  |  |  |
| Critical Integration Skill (B or C), n (%) | 13 (86.7) | 102 (28.6) | 16.3 (3.6 to 73.3) | < 0.001 |
| Traditional / Tool-oriented Skills (A, D, or E), n (%) | 2 (13.3) | 255 (71.4) | 1.0 (Reference) | - |
| Total | 15 (100.0) | 357 (100.0) |  |  |
| **Descriptive Overview: Full Response Distribution ^b^** |  |  |  |  |
| A. Technical Proficiency, n (%) | 2 (13.3) | 185 (51.8) | – | – |
| B. Critical Appraisal Skills, n (%) | 4 (26.7) | 85 (23.8) | – | – |
| C. Clinical Reasoning & Judgment, n (%) | 9 (60.0) | 17 (4.8) | – | – |
| D. Ethical & Legal Literacy, n (%) | 0 (0.0) | 10 (2.8) | – | – |
| E. Institutional Compliance, n (%) | 0 (0.0) | 60 (16.8) | – | – |

a: Calculated using Fisher’s Exact Test (two-sided) due to the small sample size in the structured training group

b: Percentages are presented for descriptive purposes. No inferential statistical tests were performed on the full five-category distribution due to limited sample size in the Structured Training Group

OR: Odds Ratio
95%CI: 95% Confidence Interval

| **Table S4 Raw contingency between structured training and Enhancement of self-reported professional competence** | | | | | |  |
| --- | --- | --- | --- | --- | --- | --- |
| Training Group | Enhancement of self-reported professional competence (Yes) (n) | Enhancement of self-reported professional competence (No) (n) | Total (n) | OR (95%CI) | *p* value* |  |
| Structured Training | 14 | 1 | 15 | 11.63 (1.90 - 124.3) | 0.0025 |  |
| No Training & Non‑structured Training Group | 195 | 162 | 357 | 1.00 (Reference) |  |  |
| Total | 209 | 163 | 372 |  |  |  |

*：The *p* value is based on Fisher’s exact test, which is used to provide an exact assessment for small cell counts. This analysis uses the single-item global self-reported competence improvement measure.
