# Supplementary material for: The “DeepSeek effect” and the adoption–integration gap of generative artificial intelligence in clinical practice: a national online convenience cross-sectional survey of academic critical care physicians in China
Source: Front Med (Lausanne). 2026 Jun 23;13:1875770. doi: 10.3389/fmed.2026.1875770 (PMC13337403; doi:10.3389/fmed.2026.1875770)
Supplement: Supplementary file 3 [file Supplementary_file_3.docx]

**Survey on the Application of Generative Artificial Intelligence in Medical Clinical Practice and Education**

**(Post-DeepSeek 2025)**

**Basic Information**

1. Your Gender: [Multiple Choice] *
   ○ Male
   ○ Female
2. Your Age: [Fill in the Blank] *
   _________________________________
3. Your Province/Region: [Fill in the Blank] *
   _________________________________
4. Your Professional Title: [Single Choice] *
   ○ Attending Physician
   ○ Associate Chief Physician
   ○ Chief Physician/Professor
5. Your Highest Educational Qualification: [Single Choice] *
   ○ Bachelor's Degree
   ○ Master's Degree
   ○ Doctoral Degree
6. Are you working in a tertiary hospital? (Single-choice)

○ Yes

○ No

**Part 1: GAI Literacy and Adoption Patterns**

1. How well do you currently understand Generative Artificial Intelligence (GAI)? [Single Choice] *
   ○ Very knowledgeable (4)
   ○ Moderately knowledgeable (3)
   ○ Slightly knowledgeable (2)
   ○ Not knowledgeable at all (1)
2. Have you participated in any training sessions or academic lectures related to GAI? [Single Choice] *
   ○ Yes
   ○ No
3. Which of the following components characterized the GAI training you participated in? [Multiple Choice]

(Applicable only if you selected "Yes" in Question 8)

○ It was a systematic or longitudinal curriculum (i.e., part of a recurring series rather than a standalone lecture).

○ It included hands-on practice or simulation sessions focusing on specific clinical tasks.

○ It provided methods for verifying the medical accuracy of GAI outputs (e.g., cross-referencing with guidelines, literature, or drug dosages).

○ It focused on identifying GAI-specific risks, such as "hallucinations," outdated knowledge, or algorithmic biases.

○ It provided guidance on integrating GAI tools into clinical workflows (e.g., electronic health record documentation, clinical decision support).

○ It covered ethical guidelines, legal liability, and institutional policies regarding GAI use in healthcare.

○ None of the above / The training focused only on basic tool functions and registration.

○ I do not recall the specific content.

1. Have you used any GAI applications (e.g., Deepseek, Kimi) in your medical-related work? [Single Choice] *
   ○ Yes
   ○ No (Please skip to Question 18)
2. In your professional opinion, which of the following is the MOST critical competency for a physician to ensure the safe and effective integration of GAI into medical work? (Single Choice)

○ Technical Proficiency: Mastery of prompt engineering and the operational features of various GAI platforms.

○ Critical Appraisal Skills: The ability to rigorously evaluate the accuracy, relevance, and reliability of AI-generated clinical content.

○ Clinical Reasoning and Judgment: The ability to discern appropriate versus inappropriate scenarios for GAI assistance within a clinical context.

○ Ethical and Legal Literacy: A deep understanding of patient privacy, informed consent, and the boundaries of professional liability when using AI.

○ Institutional Compliance: Strict adherence to standardized protocols (SOPs) and hospital-approved guidelines for AI-assisted decision-making.

1. Prior to January 2025, had you used GAI in your medical-related work? [Single Choice] *
   ○ Yes
   ○ No
2. If you currently use GAI, how frequently do you utilize it in your medical-related work? [Single Choice] *
   ○ Daily
   ○ Weekly
   ○ Monthly
   ○ Rarely (less than monthly)
3. Since January 2025, how has the frequency of your GAI usage in medical-related work changed? [Single Choice] *
   ○ Increased
   ○ Decreased (Please skip to Question 16)
   ○ Remained unchanged (Please skip to Question 16)
4. What are the main driving factors for the increased use of GAI in your medical work (clinical practice, teaching, or research) since 2024? [Please select up to 3 options and rank them in order of importance from highest to lowest] [Multiple Choice] *
   ○ Technological advancements of domestic GAI models (e.g., enhanced reasoning capabilities, optimized Chinese language support)
   ○ Improved accessibility and usability (e.g., no internet restrictions, fast response speed, reduced costs)
   ○ Strengthened data security and compliance (e.g., local deployment options, enhanced privacy protection measures)
   ○ Increased objective demand for clinical or research tasks
   ○ Professional social influences (e.g., peer recommendations, academic conference promotions, professional media coverage)
   ○ Institutional policy guidance or improved hospital informatization infrastructure
   ○ I have not increased my use of GAI since 2024
5. If you have used GAI, in which scenarios have you mainly applied it? (Multiple choices allowed) [Multiple Choice] *
   ○ Medical knowledge inquiry and updates
   ○ Preparation of medical teaching materials (e.g., courseware development, test question design)
   ○ Clinical diagnostic auxiliary support
   ○ Explanation of medical pathology or patient condition communication
   ○ Simulation of clinical consultation scenarios
   ○ Others (Please specify: ) _________________*
6. If you have used GAI, are you aware of the following characteristics of GAI? (Multiple choices allowed) [Multiple Choice] *
   ○ GAI systems are trained on datasets with a specific time cutoff, lacking post-cutoff information
   ○ General-purpose GAI models are trained on public data that may not undergo strict accuracy verification
   ○ Variations in training datasets across different GAI models can lead to differences in performance
   ○ GAI-generated content may be inferences based on existing knowledge and may contain hallucinations (fictional but plausible information)
   ○ General-purpose GAI incorporates a certain degree of randomness to ensure answer diversity, which may compromise accuracy
   ○ Different general-purpose GAI models may vary in logical rigor, information accuracy, and expression style
7. To what extent do you perceive that GAI affects physicians' professional competence?
   ○ Decrease

○ No impact

○ Increase

**Part 2: Application of GAI in Standardized Residency Training Medical Education**

1. Do you think GAI can replace traditional medical teaching methods? [Single Choice] *
   ○ Yes
   ○ No
2. Have you applied Generative Artificial Intelligence in medical education (e.g., standardized residency training, continuing medical education)? [Single Choice] *
   ○ Yes
   ○ No (Please skip to Question 26)
3. If yes, what are the main reasons driving you to use GAI in medical teaching? [Multiple Choice] *
   ○ Improve teaching efficiency and save lesson preparation time
   ○ Enhance the comprehensiveness and accuracy of teaching content
   ○ Enrich teaching methods and increase trainees' learning interest
   ○ Others (Please specify: ) _________________*
4. When using Generative Artificial Intelligence in medical education, which teaching aspects do you mainly focus on? [Multiple Choice] *
   ○ Generation of clinical simulation cases (e.g., rare disease cases, complex comorbidity scenarios)
   ○ Inquiry and supplementation of teaching-related content
   ○ Test paper generation and automatic scoring
   ○ Development of teaching materials (e.g., PPT, lesson plans, teaching handouts)
   ○ Intelligent teaching interaction and real-time feedback
   ○ Formulation of personalized teaching plans
   ○ Update and optimization of question banks
   ○ Others (Please specify: ) _________________*
5. How effective do you consider the application of GAI in medical education to be? [Single Choice] *
   ○ Highly effective
   ○ Effective
   ○ Moderately effective
   ○ Ineffective
6. What problems or challenges have you encountered when using GAI in medical education? [Multiple Choice] *
   ○ Insufficient personal experience in GAI application
   ○ Low quality of GAI-generated teaching content (e.g., inaccuracies, lack of clinical relevance)
   ○ Concerns about data privacy and information security
   ○ Insignificant improvement in teaching effectiveness
   ○ Others (Please specify: ) _________________*
7. In which aspects do you think the application of GAI in medical education needs further improvement? [Multiple Choice] *
   ○ Stability and consistency of generated content
   ○ Quality and clinical relevance of teaching content generation
   ○ Reduction of technical application barriers
   ○ Enhancement of multimodal teaching content generation (e.g., high-quality images, interactive videos)
   ○ Others (Please specify: ) _________________*
8. Please rate your level of agreement with the following statements (1=Strongly Disagree, 2=Disagree, 3=Neutral, 4=Agree, 5=Strongly Agree) [Matrix Scale Question] *

| Statements | 1 | 2 | 3 | 4 | 5 |
| --- | --- | --- | --- | --- | --- |
| GAI can improve the learning efficiency of resident physicians. | ○ | ○ | ○ | ○ | ○ |
| GAI can enhance teachers' teaching efficiency and lesson preparation quality. | ○ | ○ | ○ | ○ | ○ |
| GAI can increase resident physicians' learning interest and participation. | ○ | ○ | ○ | ○ | ○ |
| GAI can provide personalized learning support for trainees. | ○ | ○ | ○ | ○ | ○ |
| GAI helps improve the overall quality and equity of medical education. | ○ | ○ | ○ | ○ | ○ |
| I hold an optimistic attitude towards the future development of GAI in medical education. | ○ | ○ | ○ | ○ | ○ |
| I am willing to recommend colleagues to use GAI to assist in medical teaching. | ○ | ○ | ○ | ○ | ○ |
| GAI should become an official component of the future medical education system. | ○ | ○ | ○ | ○ | ○ |

1. Do you think training on GAI application should be increased for medical students or resident physicians? [Single Choice] *
   ○ Yes
   ○ No
2. Do you think training on GAI application should be increased for specialist teaching physicians or professors? [Single Choice] *
   ○ Yes
   ○ No
3. Do you think the use of GAI in medical education will affect students' critical thinking abilities? [Single Choice] *
   ○ Yes
   ○ No
4. Are you willing to recommend the use of GAI to other physicians engaged in clinical or teaching work? [Single Choice] *
   ○ Yes
   ○ No
5. The current teaching content related to GAI in medical education is sufficient. [Single Choice] *
   ○ Yes
   ○ No

**Perspectives on Ethical Teaching of GAI in Medicine**

1. Are you aware of the potential ethical issues arising from the application of GAI in medical education? [Single Choice] *
   ○ Yes
   ○ No
2. What do you think are the main aspects of ethical issues related to the application of GAI in medical education? [Multiple Choice] *
   ○ Privacy and security of medical data used for GAI content generation
   ○ Risk of academic misconduct among medical students (e.g., over-reliance on GAI for assignments)
   ○ Over-reliance of medical students on GAI, weakening independent thinking
   ○ Potential decline in medical students' own cognitive and clinical judgment abilities
   ○ Unclear attribution of responsibilities for GAI-generated medical errors
   ○ Others (Please specify: ) _________________*
3. Do you think the ethical issues of GAI in medical education have received sufficient attention from the academic community and institutions? [Single Choice] *
   ○ Yes
   ○ No
4. Artificial intelligence ethics should be an integral part of artificial intelligence education in medical schools. [Single Choice] *
   ○ Yes
   ○ No
5. Do you agree that a solid understanding of GAI ethics is an important prerequisite for the legitimate and effective use of GAI in medical work? [Single Choice] *
   ○ Yes
   ○ No

**Perspectives on GAI Application in Medical Work**

1. Clinical Auxiliary Effectiveness of Generative Artificial Intelligence: Please rate your agreement with the following statements. Rating Scale: 1=Strongly Disagree, 2=Disagree, 3=Neutral, 4=Agree, 5=Strongly Agree [Matrix Scale Question] *

| Statements | 1 | 2 | 3 | 4 | 5 |
| --- | --- | --- | --- | --- | --- |
| The differential diagnosis suggestions generated by GAI are insightful and have clinical reference value. | ○ | ○ | ○ | ○ | ○ |
| GAI can quickly organize logically clear patient condition summaries and medical reports based on patients' medical history abstracts. | ○ | ○ | ○ | ○ | ○ |
| When addressing complex drug interactions or contraindication inquiries, GAI's responses are accurate and timely. | ○ | ○ | ○ | ○ | ○ |
| The treatment plan suggestions generated by GAI are consistent with current clinical guidelines or expert consensus. | ○ | ○ | ○ | ○ | ○ |
| GAI can effectively assist me in completing the writing of various administrative approval forms or non-clinical reports. | ○ | ○ | ○ | ○ | ○ |
| Using GAI for multilingual medical record translation or professional document translation has greatly improved work efficiency. | ○ | ○ | ○ | ○ | ○ |

1. Research and Academic Support of Generative Artificial Intelligence: Please rate your agreement with the following statements. Rating Scale: 1=Strongly Disagree, 2=Disagree, 3=Neutral, 4=Agree, 5=Strongly Agree [Matrix Scale Question] *

| Statements | 1 | 2 | 3 | 4 | 5 |
| --- | --- | --- | --- | --- | --- |
| GAI can help me quickly retrieve and summarize the latest medical literature in specific fields. | ○ | ○ | ○ | ○ | ○ |
| GAI performs excellently in polishing the language expression of medical papers (English or Chinese). | ○ | ○ | ○ | ○ | ○ |
| GAI can assist me in writing statistical codes (e.g., R, Python) or organizing data analysis ideas. | ○ | ○ | ○ | ○ | ○ |
| GAI can provide valuable inspiration and frameworks for my research project design or grant proposal writing. | ○ | ○ | ○ | ○ | ○ |

1. If the results generated by GAI are inconsistent with your clinical judgment, will you trust the GAI results? [Single Choice] *
   ○ Yes
   ○ No
2. If you have not yet formed a clear clinical judgment and refer to GAI results, will you directly trust the GAI results and proceed with clinical treatment based on them? [Single Choice] *
   ○ Yes
   ○ No

**Generative Artificial Intelligence Impact Survey in Critical Care Department （Pre-DeepSeek 2024）**

1. What is your age group? (Fill-in-the-blank)

2. What is your gender? (Single-choice)

A. Male

B. Female

3. Which province in China are you from? (Fill-in-the-blank)

4. Are you working in a tertiary hospital? (Single-choice)

A. Yes

B. No

5. What is your professional title? (Single-choice)

A. Attending Physician

B. Associate Chief Physician

C. Chief Physician

6. Have you participated in GAI training? (Single-choice)

A. Yes

B. No

7. How would you rate your understanding of GAI? (Single-choice)

A. Very knowledgeable (4)

B. Moderately knowledgeable (3)

C. Slightly knowledgeable (2)

D. Not knowledgeable at all (1)

8. Do you use GAI in medical work? (Single-choice)

A. Yes

B. No (If you choose "No," please skip to Question 14)

9. If yes, how frequently do you use GAI? (Single-choice)

A. Daily

B. Weekly

C. Monthly

10. If yes, what are the main scenarios of your GAI usage? (Multiple Choice)

A. Querying Medical Knowledge

B. Preparation of presentation materials

C. Diagnosis and Therapy suggestions

D. Explanation of Medical Pathology

E. Translation

11. Do you know the following features of GAI? (Multiple Choice)

A. Trained on past data

B. Trained on public but unverified data

C. Training data choice affects performance

D. May generate speculative content

E. Set randomness reduces accuracy

12. Do you think using GAI will enhance doctors' clinical skills? (Single-choice)

A. Yes

B. No

13. What problems have you encountered when using GAI? (Multiple Choice)

A. Lack of experience with GAI

B. Low quality of content generated by GAI

C. Data privacy issues

14. What do you think are the advantages of GAI in medical education? (Multiple Choice)

A. Improve learning efficiency for residents

B. Enhance teaching efficiency for educators

C. Increase learning interest for residents

D. Provide personalized learning guidance

E. Promote fairness and raise the average level of medical education

F. Other

15. Do you think GAI training should be increased for residents? (Single-choice)

A. Yes

B. No

16. Do you think GAI training should be increased for medical teaching physicians? (Single-choice)

A. Yes

B. No

17. What is your attitude towards the future development of GAI in medical education? (Single-choice)

A. Very optimistic

B. Optimistic

C. Neutral

D. Pessimistic

18. Would you trust the results of GAI if they are inconsistent with your clinical judgment? (Single-choice)

A. Yes

B. No

19. Do you use GAI in ME-SRT? (Single-choice)

A. Yes

B. No (If you choose "No," please skip to Question 28)

20. Would you trust the results of GAI and proceed with treatment based on its recommended diagnosis before you have made your own diagnosis? (Single-choice)

A. Yes

B. No

21. What are the main reasons driving you to use GAI in medical teaching? (Multiple Choice)

A. Improve teaching efficiency and save time

B. Enhance the comprehensiveness and accuracy of teaching content

C. Enrich teaching methods and increase student interest

22. In which aspects of TSRT do you primarily use GAI? (Multiple Choice)

A. Generation of clinical simulation cases

B. Querying teaching-related content

C. Generation and grading of exams

D. Creation of teaching materials (e.g., PPT, lesson plans)

E. Intelligent teaching interaction and feedback

F. Development of teaching plans

G. Updating question banks

23. How effective do you think GAI is in medical education? (Single-choice)

A. Very effective

B. Effective

C. Moderately effective

D. Ineffective

24. What aspects of GAI application in medical education need improvement? (Multiple Choice)

A. Stability of generated content

B. Quality of generated teaching content

C. Technical difficulty of application

D. Generation of multimodal content (e.g., images and videos)

25. Does GAI use in medical education affect students' clinical thinking? (Single-choice)

A. Yes

B. No

26. Are you willing to recommend the use of GAI to other physicians? (Single-choice)

A. Yes

B. No

27. GAI should become part of medical education. (Single-choice)

A. Yes

B. No

28. Are you aware of the potential ethical issues that may arise from the use of GAI in medical education? (Single-choice)

A. Yes

B. No

29. What do you think are the main ethical issues of using GAI in medical education? (Multiple-choice question)

A. Privacy of data required for content generation

B. Academic misconduct by medical students

C. Over-reliance of medical students on GAI

D. Decline in students' cognitive and judgment abilities

E. Other

30. Do you think the ethical issues of GAI in medical education have received sufficient attention? (Single-choice)

A. Yes

B. No

31. Do you think ethics of GAI should be part of GAI education? (Single-choice)

A. Yes

B. No

32. Do you think ethics of GAI is an important prerequisite for the use of GAI? (Single-choice)

A. Yes

B. No
