# Supplementary material for: The “DeepSeek effect” and the adoption–integration gap of generative artificial intelligence in clinical practice: a national online convenience cross-sectional survey of academic critical care physicians in China
Source: Front Med (Lausanne). 2026 Jun 23;13:1875770. doi: 10.3389/fmed.2026.1875770 (PMC13337403; doi:10.3389/fmed.2026.1875770)
Supplement: Supplementary file 4 [file Supplementary_file_4.docx]

**A. Reliability and Convergent Validity**

| Constructs | Items | Loading Range | Cronbach's α | CR | AVE | MSV |
| --- | --- | --- | --- | --- | --- | --- |
| GAI in Medical Education (Q27) | 8 | 0.849–0.932 | 0.959 | 0.963 | 0.724 | 0.757 |
| GAI in Medical Work (Q38) | 6 | 0.804–0.925 | 0.957 | 0.959 | 0.796 | 0.757 |
| GAI in Research and Academic Support (Q39) | 4 | 0.852–0.907 | 0.925 | 0.927 | 0.767 | 0.700 |
| Overall Scale | 20 | 0.804–0.932 | 0.965 |  |  |  |

CITC: Corrected Item-Total Correlation; CR: Composite Reliability; AVE: Average Variance Extracted; MSV: Maximum Shared Variance

**B. Discriminant Validity Evidence**

| Metric | Value |
| --- | --- |
| Inter-factor correlation range | 0.672 – 0.805 |
| HTMT ratio range | 0.712 – 0.862 |

HTMT: Heterotrait-Monotrait ratio of correlations

### ****C. Content Validity (Full Scale, N = 41 items)****

| Metric | Value |
| --- | --- |
| Item-level CVI (I-CVI) range | 0.833 – 1.000 |
| Scale-level CVI (CVI/UA) | 0.919 |
| Scale-level CVI (CVI/Ave) | 0.986 |
| Content validity assessment | Good to excellent |

I-CVI: Item-level Content Validity Index; CVI/UA: Content Validity Index based on Universal Agreement; CVI/Ave: Content Validity Index based on the average of item-level CVIs

### ****D. Overall Model Fit Indices (CFA)****

| Index | Value | Recommended Threshold |
| --- | --- | --- |
| χ²/df | 7.948 | < 10 |
| CFI | 0.911 | ≥ 0.90 |
| TLI | 0.893 | ≥ 0.90 |
| SRMR | 0.037 | ≤ 0.08 |
| RMSEA (90% CI) | 0.137 (0.127–0.146) | ≤ 0.08 (good), ≤ 0.10 (acceptable) |

CFA: Confirmatory Factor Analysis; CFI: Comparative Fit Index; TLI: Tucker-Lewis Index; SRMR: Standardized Root Mean Square Residual; RMSEA: Root Mean Square Error of Approximation; χ²/df: Chi-square divided by degrees of freedom
